# Ovarian stem cells are always accompanied by very small embryonic-like stem cells in adult mammalian ovary

**DOI:** 10.1186/s13048-015-0200-0

**Published:** 2015-11-05

**Authors:** Deepa Bhartiya

**Affiliations:** Stem Cell Biology Department, National Institute for Research in Reproductive Health, Jehangir Merwanji Street, Parel, Mumbai, 400 012 India

**Keywords:** Stem cells, Ovarian stem cells, Very small embryonic-like stem cells, Ovary, Infertility

## Abstract

**Background:**

Existing dogma that a female is born with fixed number of eggs was challenged by the detection of stem cells in adult mammalian ovary. Data has accumulated in support of ovarian stem cells (OSCs) proliferation, maintenance in culture, formation of germ cell nests and differentiation into oocytes and primordial follicle assembly using different strategies.

**Results:**

Flow cytometry analysis identified >8 μm OSCs which are DDX1 positive and are considered equivalent to spermatogonial stem cells (SSCs) in testis. Analysis of both ovarian and testicular smears obtained after enzymatic digestion has led to the identification of an additional stem cell population termed very small embryonic-like stem cells (VSELs). VSELs and OSCs/SSCs differ from each other in their size and OCT-4 expression. VSELs express pluripotent markers including nuclear OCT-4 whereas OSCs/SSCs express cytoplasmic OCT-4 suggesting a differentiated state. VSELs can be studied by flow cytometry as small sized cells which are LIN-/CD45-/Sca-1+. We have reported 0.02 ± 0.008, 0.03 ± 0.017 and 0.08 ± 0.03 % of total cells as VSELs in normal, chemoablated and after FSH treatment to chemoablated mouse ovary.

**Conclusions:**

VSELs have remained poorly studied till now because of their very small size and rare occurrence. Spinning cells obtained after enzymatic digestion of ovarian tissue at a speed of 1000G (rather than 1200 rpm) throughout processing allows reliable detection of the VSELs by flow cytometry. VSELs exist in aged, chemoablated and non-functional ovary and providing a healthy niche to support their function offers an interesting strategy to manage infertility.

## Commentary

We read with great interest a recent review in Journal of Ovarian Research published by Silvestris et al. [1] on ovarian stem cells and how they could be used to manage infertility. They have described in a very lucid manner how the research progressed in the field and we thank them for confirming by flow cytometry that indeed DDX-1 positive ovarian stem cells (OSCs) co-express OCT-4. This confirms that the stem cells isolated from ovarian cortical tissue by Tilly’s group by flow cytometry [2] are similar to the OCT-4 positive stem cells isolated by mechanical scraping of ovary surface epithelium (OSE) by our group [3, 4]. This type of third party confirmation and validation of presence of stem cells in adult mammalian ovary is necessary to convince the scientific community and bring about a paradigm shift in the field of ovarian biology.

**Fig. 1:**
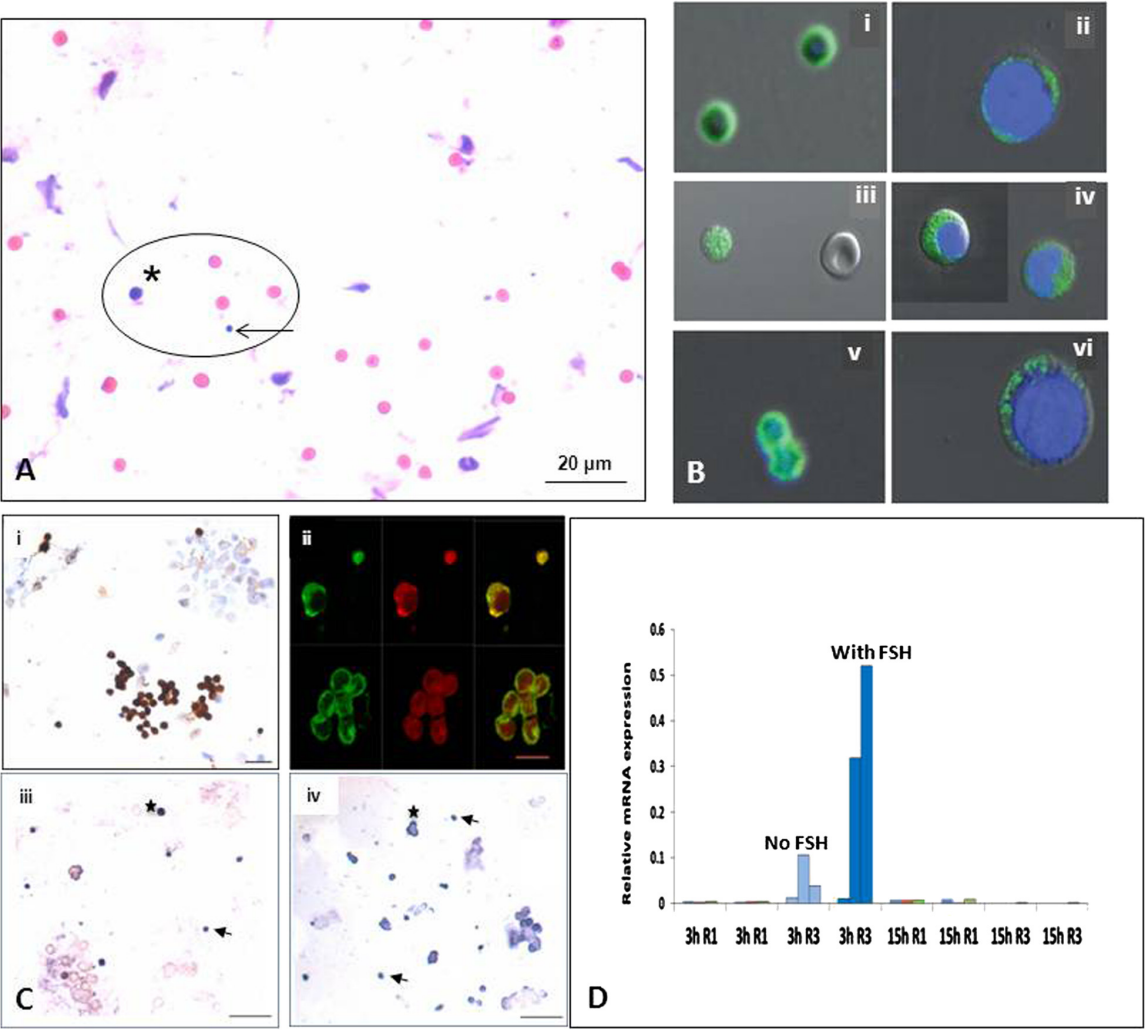
Two populations of stem cells (VSELs and OGSCs) in adult ovary. **a** H&E stained sheep OSE smear. Two distinct populations of stem cells including VSELs (*arrow*) which are smaller than the red blood cells and slightly bigger OGSCs (*asterix*) are clearly visualized even after gently scraping sheep ovary fixed overnight in neutral buffered formalin. Red blood cells and epithelial cells are also clearly visualized [4]. **b** (*i*-*vi*) Characterization of ovarian stem cells using pluripotent markers OCT-4 and SSEA-4. Smaller VSELs express nuclear OCT-4 and cell surface SSEA-4 whereas slightly bigger OGSCs express cytoplasmic OCT-4 and minimal SSEA-4. Note the VSELs do not stain with DAPI [3]. **c** (*i*) Sheep OSE smear immuno-stained with FSHR antibody. Note epithelial cells are negative whereas the stem cells exhibit distinct positive stain. (*ii*) Confocal microcopy localization of FSHR on VSELs, OGSCs and on a germ cell nest. (*iii*-*iv*) in situ hybridization of FSHR on ovarian stem cells after FSH treatment using oligoprobes specific for FSHR1 and FSHR3 respectively. Note active transcription of FSHR3 mRNA in the cytoplasm of stem cells after FSH treatment whereas FSHR1 is expressed in the stem cells and the expression is not affected by FSH treatment. **d** qRT-PCR analysis of FSHR1 and FSHR3 after 3 and 15 h of FSH treatment. Note that only FSHR3 levels are increased transiently after 3 and return to basal levels by 15 h. **c** and **d** panels show earlier published data by Patel et al. [7]. Please refer to the cited references for further details

But we have shown the co-existence of two distinct populations of stem cells in OSE including the ovary germ stem cells (OGSCs) which are the same as OSCs and a small sub-population of very small embryonic-like stem cells (VSELs) [3, 4]. The basic difference between the two populations is their size and OCT-4 expression (Fig. 1). VSELs are 3–5 μm in size and express nuclear OCT-4 whereas the OSCs are more than 8 μm in size and express cytoplasmic OCT-4. It is well known that nuclear OCT-4 is suggestive of pluripotent state of a stem cell and it is no longer required once the stem cells start to differentiate [5]. On this basis we have postulated that VSELs are the most primitive, pluripotent stem cells in the ovary and give rise to committed tissue-specific progenitors OSCs (express cytoplasmic OCT-4 and other germ cell markers) [6]. VSELs are relatively quiescent whereas the OSCs divide rapidly and form germ cell nests prior to differentiating into oocytes. Interestingly, we have also reported that ovarian stem cells express receptors for follicle stimulating hormone (FSH) and the process of asymmetric cell division (including self-renewal and giving rise to OSCs) of VSELs appears to be under the influence of FSH via alternatively spliced growth factor type 1 receptor isoform FSHR3 [7]. We have also reported similar VSELs in human and mouse testis [8, 9] and thus a similar population of VSELs in the gonads gives rise to OSCs in ovary and SSCs in testis.

The protocol discussed by Silvestris group picked up only OSCs similar to earlier published data from Tilly’s group. But flow cytometry on enzymatically isolated mouse ovarian cells and taking care to spin at 1000G (as being small in size, VSELs do not pellet at the usual 1200 rpm speed), we have reported the presence of LIN-/CD45-/SCA-1+ VSELs in normal (0.02 ± 0.008 %) and chemoablated (0.03 ± 0.017 %) adult mouse ovary [10]. Interestingly treating chemoablated mice with FSH resulted in self-renewal of VSELs as judged by an increase in their numbers by flow cytometry (0.08 ± 0.03 %), gave rise to OSCs with cytoplasmic OCT-4, formed germ cell nests and initiated differentiation into oocyte-like structures [10]. Using flow cytometry, Parte et al. [4] identified two distinct populations of stem cells in sheep OSE including VSELs (2–4 μm sized cells 1.26 ± 0.19 %) and OGSCs (4–9 μm are 6.86 ± 0.5 %). Thus the protocols to isolate OSCs published by Tilly’s group [2] and by Silvestris et al. [1] need to be modified to show that VSELs always accompany OSCs. A simple incorporation of spinning cells (collected by enzymatic isolation) at 1000G rather than the usual 1200 rpm during cell processing for flow cytometry will enable anyone to validate our claims of presence of VSELs in adult ovary.

Silvestris et al. [1] also discuss the earlier findings of Tilly’s group [11] regarding presence of germ cell markers (Oct4, MVH, Dazl, Stella, Fragilis) in bone marrow of cyclophosphamide and busulphan treated mice. Ratajczak’s group was the first to describe VSELs in 2006 [12] and various groups have observed recently that VSELs get mobilized [13–17] under stress conditions when the function of any organ in the body gets compromised. Germ cell markers in chemoablated mice bone marrow were detected by Tilly’s group [11] because VSELs were mobilized to reach the ovary in order to bring about homeostasis after ovarian function was compromised by cyclophosphamide and busulphan treatment. Furthermore, fertility restoration in chemoablated mice after BM transplantation is from the endogenous VSELs which survive chemotherapy whereas the transplanted BM only helps to improve the niche thus explaining the observations of Niikura et al. [18] that transplanting GFP+ BM in chemoablated mice resulted in non-GFP pups.

It is very true that protocols for the use of cryopreserved cortical tissue for fertility preservation are not yet perfect and a risk of re-introducing malignant cells exists. A total of 37 babies have been born by transplanting cortical tissue in cancer survivors [19] and Demeestere et al. [20] recently reported spontaneous pregnancy and live-birth from ovarian cortical tissue cryopreserved for more than a decade; but the true source of the oocytes in these cases (whether it is the transplanted cortical tissue or the non-functional ovary) that results in the babies is still not clear and has been recently discussed by us [21]. Simultaneously, efforts are ongoing to differentiate embryonic stem cells and induced pluripotent stem cells into primordial germ cells in mice [22] as well as in humans [23]. Obtaining PGCs from ES cells is crucial as they exhibit an inherent potential to spontaneously differentiate into oocytes [24]. While ES cells during the course of development are obtained from inner cell mass of developing blastocyst, PGCs are relatively mature and arise from the epiblast stage embryo. ES cells and PGCs basically differ from each other in their epigenetic status since PGCs (during their migration along the dorsal mesentery to reach the gonadal ridge) undergo erasure of imprinted genes. It is very difficult to mimic this epigenetic maturity of PGCs in vitro starting with ES cells and thus the roadblock in obtaining ‘synthetic gametes’ from hES cells to cure infertility. On the other hand, VSELs are postulated to be equivalent to the PGCs and we have reported that they spontaneously differentiate into oocytes in vitro [3, 10] and also sperm [25]. This understanding and better potential of VSELs over ES cells to form gametes in vitro was recently discussed [26] and needs to be appreciated by the scientific community. Vassena et al. [27] have also recently confused between OSCs and VSELs.

For treating infertility–the million dollar question is whether we need to treat and administer more stem cells in the non-functional ovary or improve the microenvironment for stem cells (that may be existing) to function properly! Various groups have suggested that decline of ovarian function with age is because the niche gets compromised and is unable to support stem cells differentiation into oocytes. On transplanting stem cells from aged ovary into a young ovary resulted in normal oocytes formation [18]. We found stem cells in the ovarian tissue obtained from a 60 years old woman which spontaneously differentiated into oocyte-like structures on putting in culture (where unfavorable factors in vivo are overcome). Similarly we have also reported that culture of both (i) chemoablated intact mouse ovary and (ii) the OSE cells obtained by enzymatic isolation results in spontaneous differentiation of oocyte-like structures [10]. Thus it is only required to improve the microenvironment for stem cells to function and thereby cure infertility, menopause etc. Transplanting Sertoli cells and bone marrow derived mesenchymal cells independently in chemoablated testis restored spermatogenesis [9]. Many groups have reported improved gonadal function and live pups by transplanting mesenchymal cells from various sources into chemoablated gonads. Caicki et al. [28] and Lai et al. [29] transplanted mesenchymal cells expanded from adipose tissue and endometrium respectively into chemoablated gonads and obtained live births. It is postulated that transplanted mesenchymal cells help improve the niche and enable VSELs to regenerate chemoablated gonads.

To conclude, adult mammalian ovary harbors VSELs and OSCs just like VSELs and SSCs in the testis. Endogenous VSELs can be targeted to regenerate non-functional gonads and thus offer an interesting alternative strategy to cure infertility.

## Abbreviations

OGSCs: Ovarian germ stem cells
OSCs: Oogonial stem cells
OSE: Ovary surface epithelium
PGCs: Primordial germ cells
SSCs: Spermatogonial stem cells
VSELs: Very small embryonic-like stem cells
