# Correction to: ovarian stem cells are always accompanied by very small embryonic-like stem cells in adult mammalian ovary

**DOI:** 10.1186/s13048-023-01254-7

**Published:** 2023-08-14

**Authors:** Deepa Bhartiya

**Affiliations:** Stem Cell Biology Department, National Institute for Research in Reproductive Health, Jehangir Merwanji Street, Parel, Mumbai, 400 012 India

Correction: J Ovarian Res 8:70 (2015)

10.1186/s13048-015-0200-0

Following publication of the original article [1], please note that Figs. 1A and C are composites prepared by putting together various fields as these cells are spread far apart on the slides.
